# Prevalence and Determinants of Peripheral Microvascular Endothelial Dysfunction in Rheumatoid Arthritis Patients: A Multicenter Cross-Sectional Study

**DOI:** 10.1155/2018/6548715

**Published:** 2018-02-01

**Authors:** Gian Luca Erre, Matteo Piga, Anna Laura Fedele, Silvia Mura, Alessandra Piras, Maria Luisa Cadoni, Ignazio Cangemi, Martina Dessi, Gabriele Di Sante, Barbara Tolusso, Elisa Gremese, Alberto Cauli, Arduino Aleksander Mangoni, Pier Sergio Saba, Ciriaco Carru, Gianfranco Ferraccioli, Alessandro Mathieu, Giuseppe Passiu

**Affiliations:** ^1^UOC di Reumatologia, Azienda Ospedaliero-Universitaria di Sassari, Sassari, Italy; ^2^UOC di Reumatologia, Policlinico Universitario di Monserrato, Cagliari, Italy; ^3^UOC di Reumatologia, Fondazione Policlinico Universitario A. Gemelli-Catholic University of the Sacred Heart, Roma, Italy; ^4^Department of Clinical Pharmacology, Flinders University and Flinders Medical Centre, Adelaide, Australia; ^5^UO di Cardiologia, Azienda Ospedaliero-Universitaria di Sassari, Sassari, Italy; ^6^Dipartimento di Scienze Biomediche, Università degli Studi di Sassari, Sassari, Italy

## Abstract

**Objectives:**

To define the prevalence and determinants of peripheral microvascular endothelial dysfunction (ED) in a large series of rheumatoid arthritis (RA) patients free of previous cardiovascular events.

**Materials and Methods:**

Data from 874 RA patients enrolled in the EDRA study (Endothelial Dysfunction Evaluation for Coronary Heart Disease Risk Estimation in Rheumatoid Arthritis—ClinicalTrials.gov: NCT02341066) were analyzed. Log-transformed reactive hyperemia index (Ln-RHI) was evaluated by peripheral arterial tonometry (PAT) using the EndoPAT2000 device: values of Ln-RHI < 0.51 were considered indicative of peripheral ED.

**Results:**

Peripheral microvascular ED was documented in one-third of RA patients (33.5%); in multiple logistic regression analysis, ACPA negativity and higher triglycerides concentrations were independently associated with the presence of peripheral ED [OR (95% CI) = 1.708 (1.218–2.396), *p* < 0.01 and OR (95% CI) = 1.005 (1.002–1.009), *p* < 0.01, respectively]. Multiple regression analysis showed a positive correlation between Ln-RHI values and systolic blood pressure and HDL cholesterol levels; furthermore, higher values of Ln-RHI were associated with ACPA positivity, while smoking habit was associated with lower Ln-RHI values.

**Conclusions:**

This study demonstrates for the first time a high prevalence of peripheral microvascular ED in patients with RA free of previous cardiovascular events that appear to be only partially driven by traditional cardiovascular risk factors. The association between ACPA negativity and ED warrants further exploration.

## 1. Introduction

Rheumatoid arthritis (RA) is a chronic progressive disease associated with systemic inflammation that mainly affects synovial joints leading to tissues destruction, disability, and excess of mortality.

RA patients suffer a significantly reduced life expectancy (by 3 to 18 years) with respect to the general population with a standardized mortality ratio ranging from 1.2 to 2.7 [1]. This excess of mortality in RA patients has not changed over the past 20 years [2].

About one-third of premature deaths in RA are due to cardiovascular disease (CVD) [3], primarily coronary heart disease (CHD). Mortality risk for CHD in RA patients has been estimated to be >50% higher than the general population [4]. Moreover, unlike the general population, global CV mortality in RA has not appeared to have fallen over time [5] despite relevant improvements in early diagnosis and treatment.

This excess of CHD is not fully explained by the higher prevalence of traditional CV risk factors (smoking, dyslipidemia, hypertension, and diabetes) in RA patients with respect to the general population [6, 7]. Thus, it is conceivable that other nonconventional risk factors, likely related to systemic inflammatory RA burden, may be involved in chronic vascular atherosclerotic damage ultimately resulting in CHD and global cardiovascular disease. Therefore, there is an urgent need to develop novel CV risk scores encompassing novel risk factors to provide a more reliable estimate of CV risk in RA.

A significant impairment of both the compliance of the central arterial system, termed arterial stiffness, and the endothelial function was frequently reported in the RA population [8, 9].

Endothelial dysfunction (ED), the earliest pathological alteration of the arterial wall in atherosclerosis, is a measure of impaired nitric oxide (NO) synthesis and availability, hence a reduced vasodilatory and atheroprotective function. ED is associated with virtually all known CV risk factors [10] and independently predicts the risk of future CV events in the general population [11]. Therefore, measuring ED should be seen as a valuable tool for CV risk stratification, over and above established scoring systems such as the Framingham Risk Score (FRS).

However, a poor correlation between peripheral microvascular and macrovascular endothelial function has been reported in RA patients [12, 13]. Furthermore, until now, very little attention has been paid to the assessment of microvascular ED and its associations with a comprehensive panel of clinical and demographic factors in this population.

Microvascular ED can be evaluated noninvasively by laser Doppler imaging (combined with iontophoresis of acetylcholine and sodium nitroprusside) to the forearm and by pulse amplitude tonometry (PAT) of the small digital artery.

PAT has recently gained attention as a useful tool to measure peripheral microvascular ED in an outpatient setting because it is a simple, rapid, noninvasive, and operator-independent technique. Briefly, PAT measures reactive hyperemia of the small digital artery (sa-RH) after an ischemic stimulus in the forearm. PAT shows high grade of correlation with gold standard measures of coronary ED [14]. Moreover, ED determination by PAT is related to CV risk factors in the Framingham cohort [15] and has proven to predict CHD [16] and future CV events [17, 18].

To the best of our knowledge, the available evidence on peripheral microvascular ED evaluated by PAT in RA is limited to a single study enrolling 55 patients [19]. In this study, ED did not show any significant associations with conventional cardiovascular risk factors and its prevalence was not assessed.

Therefore, we sought to determine prevalence and determinants of peripheral microvascular ED by PAT in a large RA population free of previous overt CVD.

## 2. Patients and Methods

### 2.1. Patient Selection

We reported data on the peripheral microvascular endothelial function of 874 RA patients aged 45–85 years without evidence of clinically overt cardiovascular disease. Patients were prospectively enrolled in the multicenter 3-year prospective cohort EDRA study (Endothelial Dysfunction Evaluation for Coronary Heart Disease Risk Estimation in Rheumatoid Arthritis study (EDRA) – ClinicalTrials.gov: NCT02341066) between October 2015 and July 2017. The EDRA study was approved by the Azienda ASL 1 of Sassari (Italy) Institutional Review Board (2126/CE-2015) and was conducted in accordance with the guidelines of our institutional ethics committees and the Declaration of Helsinki. Written informed consent was obtained from each patient before participation.

The EDRA study aimed to evaluate the incremental value of ED, assessed by Endo-PAT, when added to the FRS, in predicting CHD events in a cohort of 3000 RA patients. Inclusion criteria were (a) men and women aged > 45 and <84 years, and (b) RA as defined by the ACR/EULAR 2010 RA classification criteria [20]. Exclusion criteria were (a) previous CV or cerebrovascular events (acute coronary syndrome, stable angina, stroke, interventional procedures, carotid endarterectomy, and symptomatic peripheral artery ischemia), (b) abnormal ECG at rest, (c) sign or symptoms of autonomic nervous system dysfunction, (d) serious infections in the previous 6 months, (e) concomitant severe illness (overt hepatic insufficiency and renal disease, GFR < 30 ml/min, Cockroft-Gault formula), (f) recent diagnosis of cancer, and (g) pregnancy.

The EDRA study is ongoing at three sites, two regional hospitals (site 1, Sassari; site 2, Cagliari) and one central (site 3, Roma) hospital. A total of 270 patients (100 at site 1, 150 at site 2, and 20 at site 3) denied informed consent; 5 Endo-PAT tests were not reliable to low signal; 3 Endo-PAT tests were not correctly performed due to severe digital deformities related to RA. A total of 874 Endo-PAT tests where then available for the subsequent analysis.

### 2.2. Baseline Characteristics

The following baseline characteristics were registered on the same day of PAT assessment: hypertension (blood pressure ≥ 140/90 mmHg or treatment with antihypertensive medications), diabetes mellitus (patient history and/or treatment with insulin or oral hypoglycaemic agents), family history of CHD in first-degree relatives, and smoking habit. We also performed a 12-lead conventional ECG. Lipid profile data (total HDL and LDL cholesterol and triglycerides concentrations, all expressed in mg/dL) collected within 3 months prior to the study as routine clinical practice were registered. To assess potential correlations between the RA phenotype and peripheral ED, the following disease specific scores, disease descriptors, and treatment data were recorded and collected: steroid treatment; cumulative steroid dose in the last month; treatment with synthetic or biological disease-modifying antirheumatic drugs (DMARDs); number of swollen joints; number of tender joints; C-reactive protein (CRP) concentrations, mg/dL; erythrocyte sedimentation rate (ESR), mm/h; Disease Activity Score-28 (DAS-28); Health Assessment Questionnaire (HAQ); positivity for IgM-rheumatoid factor (IgM-RF); and anticitrullinated cyclic peptide antibodies (ACPA).

### 2.3. Endo-PAT

Patients were studied in a fasting state. Antihypertensive drugs were withheld on the study day. Finger probes consisting of thimble a-shaped sensor cap which register pulsatile volume changes were placed on the middle finger of each subject's hand. Changes in digital pulse amplitude were sensed by pressure transducers, filtered, amplified, and then recorded for further analysis by the EndoPAT 2000 device (Itamar Medical Inc., Caesarea, Israel).

After a 5 min baseline measurement, arterial flow in the brachial artery was interrupted by a cuff placed on a proximal forearm and inflated to 200 mmHg or 60 mmHg above baseline systolic blood pressure for 5 min. Then, the cuff was deflated and the digital pulse amplitude was recorded for a further 6 min. The ratio of the postischemic pulse amplitude signal compared with baseline was calculated, normalized for the baseline signal, and indexed to the contralateral one. The log-transformed ratio, expressed as Ln-RHI, reflects the small artery reactive hyperemia. Bonetti et al. reported that a RHI value of < 1.67 (corresponding to a Ln-RHI < 0.51) had a sensitivity of 82%, a specificity of 77%, and an AUC of 0.82 for diagnosing coronary ED [14]. Therefore, we used a Ln-RHI cutoff value <0.51 to define the presence of a significant ED.

### 2.4. Statistical Analysis

Continuous variables are presented as mean ± SD whereas categorical variables are presented as frequencies (*n*) or percentages (%). Variables with a nonnormal distribution were log-transformed for further analysis. Univariate association was tested by Pearson correlation analysis or by Mann–Whitney *U* test analysis. Multiple linear regression analysis was performed to analyze linear correlation between predictors and Ln-RHI. The variables related to ED with a *p* < 0.05 at the univariate logistic regression analysis entered into a multivariate logistic regression model in which the “presence of ED” was the variable to be explained. Results are expressed as the odds ratio (OR) and 95% confidence interval (95% CI). Analyses were performed using SPSS (Version 20, SPSS Inc., Chicago, IL, USA). A *p* < 0.05 was considered statistically significant.

## 3. Results

The demographic, cardiovascular, and biochemical characteristics of RA patients are shown in Tables 1 and 2.

The median Ln-RHI value for the overall RA population was 0.67 ± 0.3. Men had a trend towards lower median Ln-RHI values than women (0.63 ± 0.3 versus 0.68 ± 0.3, *p* = 0.055). One-third (33.5%) of RA patients exhibited peripheral ED (Ln-RHI < 0.51) (Table 3).

In a bivariate correlation analysis, Ln-RHI was positively, albeit weakly, correlated with systolic blood pressure, ACPA positivity, and HDL cholesterol concentrations. RHI was also inversely correlated with smoking habit (Table 4). In multiple regression analyses, systolic blood pressure, HDL cholesterol concentrations, and ACPA positivity remained independently associated with higher Ln-RHI whereas smoking was independently associated with lower Ln-RHI (Table 4).

After stratification for the ACPA status, none of the previously identified factors was independently associated with Ln-RHI in ACPA-negative patients. On the other hand, the effect of systolic blood pressure (B coefficient 0.002; 95% CI 0.001–0.004) and smoking (B coefficient −0.088; 95% CI from −0.162 to −0.015) was confirmed in ACPA-positive patients whereas disease duration (B coefficient per year −0.000; 95% CI from −0.001 to −0.000) was independently associated with lower Ln-RHI in this subgroup (Supplementary file (available here)). However, these findings cannot be considered reliable as the study sample was not designed for this kind of subanalysis.

RA patients with pathological Ln-RHI values had lower systolic blood pressure and diastolic blood pressure, higher levels of triglycerides, a higher BMI, and a longer disease duration (Table 5). RA patients with ED were in higher percentage smokers compared to patients with normal Ln-RHI values. A higher frequency of ACPA negativity was found among patients with ED, compared to patients without ED (Table 5).

In logistic regression analysis, ACPA negativity and higher serum triglyceride concentrations were independently associated with the presence of peripheral ED, whereas higher systolic blood pressure values were modestly associated with a reduced risk of ED (Table 5). In multiple logistic regression analysis, ACPA negativity was the factor that was most strongly associated with the presence of peripheral ED [OR (95% IC) = 1.708 (1.218–2.396); *p* < 0.01] even after adjustment for smoking habit (Table 5).

Other than expected, we found no significant relationship between measures of inflammatory burden (ESR and CRP), disease severity (DAS28 and HAQ), RA treatment patterns (use and dosage of steroids, use of synthetic DMARDs, and use of biological DMARDs), and microvascular reactivity (data not shown).

## 4. Discussion

Despite an increasing number of studies assessing ED in RA, mostly based on the measurement of flow-mediated dilatation (FMD) of the brachial artery [12], the prevalence and the factors associated with its presence remain largely unknown.

To our knowledge, this is the first study assessing microvascular endothelial function by PAT in a large series of prospectively enrolled RA patients without previous cardiovascular events. Our results are of interest in basic research and, possibly, in clinical practice.

We used PAT technology, instead of brachial FMD, due to its independence of operator, easy of use, and simplicity in implementation in our outpatient clinics. Furthermore, although largely based on the same physiological mechanism (endothelium-dependent vasodilation), peripheral microvascular and macrovascular endothelial function are shown to be largely independent from each other in RA [13]. No significant correlation between FMD and PAT, after adjustment for confounders, was reported in two large community studies [21, 22]. Similarly, a small study found no association between FMD and PAT in SLE, an autoimmune rheumatic disease, sharing with RA some common pathogenetic mechanisms and systemic features [23].

This lack of concordance between PAT and FMD may suggest distinct pathophysiologies in conductance vessels and digital microvascular bed.

The primary novel finding of this study is that up to a third of RA patients free of previous cardiovascular events exhibit significant peripheral microvascular ED, as demonstrated by an impairment of microvascular hyperemic response.

Of note, lower peripheral microvascular vasodilatory function has proven to significantly predict the risk of future coronary events [16, 24] in the general population: a relative risk of 0.76 (95% CI 0.65–0.88) per each 0.1 increase of Ln-RHI was reported from a meta-analysis of 6 studies reporting prospectively collected data on cardiovascular outcomes [18]. Therefore, it is conceivable that a high prevalence of peripheral microvascular ED might translate into accelerated atherosclerosis and increased risk of future cardiovascular events also in RA population.

In the present investigation and consistent with previous reports, only a weak correlation was observed between microvascular reactive hyperemia and major conventional cardiovascular risk factors, such as advancing age and gender [14, 15].

Unexpectedly, Ln-RHI correlated positively with systolic blood pressure; the same result was obtained from 3 large community-based studies involving in total over 7500 subjects [15, 22, 25]. The mechanism behind this association is not well understood, but the possibility that factors related to blood pressure-dependent brachial artery blood flow may significantly impact on RHI cannot be ruled out. Indeed, Lee et al. demonstrated a close relationship between basal blood flow in the brachial artery and reactive hyperemia-induced changes in the digital artery diameter and flow velocity [26]. It could be hypothesized that a higher basal pulse amplitude results into a higher microvascular reactivity. However, further research in experimental models and humans is warranted to address this issue.

Total cholesterol and LDL cholesterol concentrations were not associated with impaired microvascular reactivity in our series of RA patients. However, it should be taken into account that RA patients exhibit significantly lower concentrations of these lipid fractions with respect to the general population [27]. Accordingly, the increased CVD risk related to RA has shown to be paradoxically associated to relatively low cholesterol concentrations, a phenomenon known as the “lipid paradox” that has been related to systemic inflammation [28]. On the contrary, HDL cholesterol concentrations were positively associated with higher microvascular reactivity in this study. Of note, HDL cholesterol has shown to be protective on the endothelium, increasing the endothelium nitric oxide synthase- (eNOS-) mediated production of the vasodilator NO [29].

Similar to our findings, Ferré et al. in a series of 816 subjects at intermediate to high cardiovascular risk reported that HDL cholesterol was the main determinant of microvascular reactivity [30].

Similarly to previous reports [31], hypertriglyceridemia and smoking were inversely associated with RHI in our population. Smoking habit negatively impacts on macro and microvascular bed vasodilatory capacity trough impaired eNOS-related NO availability. Therefore, pharmacological and nonpharmacological measures aimed at smoking cessation, increasing HDL cholesterol, and reducing triglycerides concentrations may have a significant positive impact on peripheral ED in RA.

Failure of other traditional cardiovascular factors in showing significant associations with Ln-RHI suggests that further “unexplained” factors, such as systemic inflammation, may drive microvascular reactivity in RA.

However, in this study, other than expected, we were not able to find significant correlations between measure of systemic inflammation (CRP, ESR, and DAS-28) and digital hyperemic response.

Similarly, in age- and sex-adjusted analyses, no significant relationship was demonstrated between CRP and PAT ratio in the large cross-sectional study by Hamburg et al. from the Framingham cohort [15].

Therefore, interactions between inflammation, conventional cardiovascular risk factors, and vascular function, more than inflammation alone, may explain impairment of microvascular reactivity in RA patients.

ACPA and RF are the most characteristic RA-specific autoantibodies. ACPA-positive RA patients differ from seronegative ones in genetic and environmental risk factors and response to treatment. The presence of a significant relationship between autoantibody positivity, including ACPA, and ED accelerated atherosclerosis, CVD incidence, and mortality in RA is a matter of debate.

A significant process of protein citrullination has been demonstrated into atheroma suggesting that ACPA positivity mirrors ongoing accelerated atherosclerosis. However, ACPA positivity was not related to carotid intima-media thickness in a cross-sectional controlled study in RA patients [32]. Similarly, in a prospective study looking at identifying parameters associated with the development of subclinical atherosclerosis in a very early arthritis cohort, ACPA positivity was associated with thinner carotid intima-media thickness after 7 years of follow-up [33]. By contrast, available data on ACPA status and microvascular dysfunction in a previous study [19] reported significantly lower mean RHI values in 33 ACPA-positive patients when compared to 22 ACPA-negative RA patients (RHI = 1.78 versus 2.19, respectively, *p* = 0.008) [17].

A large study including 937 RA patients reported an association between ACPA positivity and risk of CHD (OR 2.58, 1.17–5.65) [34] while other studies did not observe a significant relationship between ACPA status and CVD incidence in RA population [35, 36]. In the Women Health Initiative study following postmenopausal women with self-reported RA, ACPA positivity was not significantly associated with higher rates of incident CVD morbidity or mortality. Accordingly, RF, but not ACPA, was related to CVD mortality in a longitudinal observational study of US veterans with RA [37]. Furthermore, in a recent Canadian prospective multicentre inception cohort study of 2626 RA patients, cardiovascular event rates in seropositive versus seronegative subjects were not significantly different. Although seropositivity was not associated with incident cardiovascular events in multivariable Cox regression models, the calculated relative risk was 0.81, suggesting a potential protective effect of ACPA positivity towards cardiovascular disease [38].

Collectively taken, these data do not clearly support a significant correlation between ACPA positivity and atherosclerotic CVD burden in the RA population. Even considering this scenario, our data showing a significant negative association between ACPA status and microvascular peripheral ED are unexpected and a firm biologically plausible explanation is lacking. Therefore, further studies are needed to explore whether ACPA may act (in)directly to prevent ED or merely associate to specific factors involved in endothelial function in RA patients.

The main limitation of our work was that RA patients were under treatment for the control of cardiovascular risk factors at the moment of PAT evaluation. Nevertheless, the treatment regimen had no significant effect on PAT measures in multiple logistic regression analysis (data not shown).

Moreover, the observational design of this study did not enable us to make conclusive considerations about cause-and-effect relationship and direction of association between microvascular function and covariates.

Finally, we did not use a comparator, for example, healthy subjects. However, although the comparison between patients and the general population could be of general interest for the understanding of accelerated atherosclerosis in RA, the purpose of the EDRA study was to investigate ED as a risk factor for new CV events in RA, and therefore it did not include a control population.

## 5. Conclusions

This is the first study to show that small artery reactive hyperaemia as measured by PAT is reduced in up to a third of RA patients free of previous cardiovascular events. HDL cholesterol and systolic blood pressure were positively associated with RHI, whereas smoking, ACPA negativity, and triglycerides concentrations were inversely associated with RHI. Furthermore, systemic inflammation per se does not appear to influence peripheral ED in the RA population. The negative association between ACPA and peripheral ED warrants further exploration in prospective studies.

We expect that the prospective data from the EDRA study will offer more conclusive data on the clinical relevance of microvascular ED evaluation by PAT in improving the prediction of CVD in the RA population.

## Figures and Tables

**Table 1:** Demographics and cardiovascular risk factors of RA population.

	Overall *n* = 874	ED *n* = 293	No ED *n* = 581	*p* value
Age (yrs)	60.9 ± 9	61.3 ± 9	60.7 ± 9	ns
Smoking habit (%)	20.8	25.2	18.6	0.02
Hypertension (%)	44	48	43	ns
Systolic blood pressure (mmHg)	127.8 ± 16	125.4 ± 15	129 ± 17	0.002
Diastolic blood pressure (mmHg)	76.9 ± 9	75.8 ± 8	77.4 ± 10	0.02
BMI (kg/m^2^)	28.3 ± 33	29.4 ± 32	27.8 ± 34	ns
Dyslipidemia (%)	32.5	28.3	34.7	ns
Diabetes (%)	7.4	8.3	6.9	ns
Total cholesterol (mg/dL)	205.7 ± 36	207.8 ± 36	204.7 ± 36	ns
HDL cholesterol (mg/dL)	61.3 ± 15	60.2 ± 16	61.8 ± 15	ns
LDL cholesterol (mg/dL)	124.5 ± 31	126.2 ± 31	123.7 ± 32	ns
Triglycerides (mg/dL)	98 ± 44	103.7 ± 48	95.3 ± 42	0.01

ns: not significant.

**Table 2:** RA descriptors.

	Overall *n* = 874	ED *n* = 293	No ED *n* = 581	*p* value
Disease duration (months)	131.8 ± 116	141.1 ± 116	127 ± 115	ns
ACPA positivity (%)	62.8	55.9	66.2	0.009
IgM-RF positivity (%)	67	62.8	69	ns
ESR (mm/h)	26.4 ± 20	25.9 ± 21	26.7 ± 20	ns
CRP (mg/dL)	0.59 ± 0.9	0.56 ± 0.7	0.61 ± 0.9	ns
DAS-28	3.53 ± 1.3	3.51 ± 1.2	3.52 ± 1.3	ns
HAQ	0.75 ± 0.6	0.79 ± 0.7	0.73 ± 0.6	ns
Steroid use (%)	37.6	38.4	37.2	ns
Steroid dose (mg/day)	2.9 ± 3.9	3 ± 4.6	2.8 ± 3.6	ns
Cumulative steroid dose (mg/month)	87 ± 119	91.2 ± 139	84.9 ± 108	ns
NSAID use (%)	23.4	25.1	22.6	ns
DMARD use (%)	78.6	78.8	78.5	ns
TNFi use (%)	28.7	29.1	28.5	ns
Tocilizumab use (%)	7.2	7.2	7.1	ns
Abatacept use (%)	5.1	5.1	5.1	ns
Rituximab use (%)	2.4	2.4	2.4	ns

ns: not significant.

**Table 3:** Peripheral endothelial dysfunction by EndoPAT2000 in RA population.

	Overall *n* = 874	Males *n* = 213	Females *n* = 661	*p* value
Ln-RHI	0.67 ± 0.32	0.63 ± 0.31	0.68 ± 0.33	0.055
Ln-RHI ≤ 0.51	293 (33.5)	35.2	33	ns
Ln-RHI ≤ 0.44 (1Q)	223 (25.5)	25.6	25.4	ns

Values are expressed as median ± 1SD; Ln-RHI: logarithmic reactive hyperemia index; 1Q: first quartile.

**Table 4:** Independent determinants of Ln-RHI.

Independent variable	Bivariate correlation Spearman rho	Univariate linear regression B coefficient (95% IC)	Multiple linear regression B coefficient (95% IC)
Systolic blood pressure (mmHg)	0.10^∗^	0.002 (0.001–0.003)^∗^	0.003 (0.001–0.004)^∗^
ACPA positivity	0.10^∗^	0.054 (0.006–0.102)^∧^	0.089 (0.035–0.144)^∗^
Smoke habit	−0.10^∗^	−0.078 (from −0.132 to −0.025)^∗^	−0.085 (−0.153–0.017)^∧^
Dyslipidemia	0.08^∧^	0.060 (0.008–0.111)^∧^	
BMI	−0.086^∧^		
HDL cholesterol (mg/dL)	0.073^∗^	0.001 (0.000–0.003)^∧^	0.002 (0.000–0.004)^∧^
Triglycerides (mg/dL)	−0.07^∧^	−0.001 (from −0.001 to –0.000)^∧^	
Disease duration	−0.06^∧^		

A linear regression for multiple variables (stepwise method) was performed including into the model variables showing significant association (*p* < 0.05) with the dependent variable Ln-RHI at the univariate regression analysis. Age and gender were forced in the model. ^∧^*p* < 0.05, ^∗^*p* < 0.01.

**Table 5:** Independent determinants of peripheral ED.

	ED *n* = 293	No ED *n* = 581	Binary logistic analysis OR (95% IC)	Multivariate logistic analysis OR (95% IC)	Cox and Snell *R*^2^
Systolic blood pressure (mmHg)	125.4 ± 15.5	129 ± 17.1	0.98 (0.97–0.99)^a^^∗^	0.98 (0.97–0.99)^a^^∗^	0.04
Triglycerides (mg/dL)	103.7 ± 48.2	95.4 ± 42.7	1.004 (1.001–1.007)^b^^∧^	1.005 (1.002–1.009)^b^^∗^	
ACPA negativity [*n* (%)]	44.1	33.8	1.546 (1.126–2.122)^∗^	1.708 (1.218–2.396)^∗^	
Smoking habit [*n* (%)]	25.2	18.6	1.468 (1.047–2.059)^∧^	—	
Diastolic blood pressure (mmHg)	75.9 ± 8.9	77.4 ± 10.2	0.98 (0.97–0.99)^∧^	—	
BMI (kg/m^2^)	29.5 ± 32.5	27.9 ± 34.4	—	—	
Disease duration (months)	141.2 ± 116.8	127.1 ± 115.8	—	—	

Odds ratio (OR) is based on the risk of the dependent variable (low Ln-RHI) given the presence of the independent variable. 95% CI: 95% confidence interval. Multivariate logistic analysis with backward logistic regression method has been performed including in the model variables showing significant (*p* < 0.05) association with the dependent variable (low Ln-RHI) at the binary logistic analysis. ^∗^*p* < 0.01; ^∧^*p* < 0.05; ^a^per mmHg; ^b^per mg/dL.

## Supplementary Materials

Supplementary Materials Table S1: Independent determinants of Ln-RHI according to ACPA status. Table S2: Independent determinants of peripheral ED according to ACPA status.
