# Supplementary material for: Prevalence and Determinants of Peripheral Microvascular Endothelial Dysfunction in Rheumatoid Arthritis Patients: A Multicenter Cross-Sectional Study
Source: Mediators Inflamm. 2018 Feb 1;2018:6548715. doi: 10.1155/2018/6548715 (PMC5816852; doi:10.1155/2018/6548715)
Supplement: Supplementary Materials — Table S1: Independent determinants of Ln-RHI according to ACPA status. Table S2: Independent determinants of peripheral ED according to ACPA status. [file 6548715.f1.doc]

**Supplementary file**

**Table Is. Independent determinants of Ln-RHI according to ACPA status**

| Independent variable | Bivariate  correlation  Spearman rho | Univariate linear regression  B coefficient (95%IC) | Multiple linear regression  B coefficient (95%IC) |
| --- | --- | --- | --- |
| *ACPA negative* |  |  |  |
| Dyslipidemia | 0.14^ | - | - |
|  |  |  |  |
| *ACPA positive* |  |  |  |
| Systolic blood pressure, mm Hg | 0.14* | 0.002(0.001-0.004)^ | 0.002(0.001-0.004)^ |
| Smoke habit | -0.10* | -0.08(-0.154--0.007)^ | -0.088(-0.162--0.015)^ |
| Disease duration | -0.13* | 0.000(-0.001-0.000)^ | 0.000(-0.001-0.000)* |
| HDL cholesterol, mg/dL | 0.09^ | - | - |
| Diastolic blood pressure, mm Hg | 0.09^ | - | - |

A linear regression for multiple variables (stepwise method) was performed including into the models variables showing significant association (p<0.05) with the dependent variable Ln-RHI at the univariate regression analysis.

Age and gender were forced in the model. ^*p<*0.05; *p<0.01; **p<0.001;

**Table IIs. Independent determinants of peripheral ED according to ACPA status**

|  | ED | No ED | Binary logistic analysis OR(95%IC) | Multivariate logistic  analysis OR(95%IC) | Cox and Snell  R^2^ |
| --- | --- | --- | --- | --- | --- |
| *ACPA negative* |  |  |  |  |  |
| - |  |  | - | - | - |
|  |  |  |  |  |  |
| *ACPA positive* |  |  |  |  |  |
| Diastolic blood pressure, mmHg | 75±9 | 77.2±10 | 0.978(0.958-0.998)^ | - |  |
| Systolic blood pressure, mmHg | 123.4±15 | 128.3±17 | 0.982(0.969-0.994)* | 0.979(0.966-0.993)* |  |
| Triglycerides, mg/dL | 109.3±54 | 96.9±41 | 1.006(1.001-1.010)^ | 1.006(1.002-1.011)* | 0.04 |

Odds ratio (OR) is based on the risk of the dependent variable (low LnRHI), given the presence of the independent variable. 95% CI 95% confidence interval. Multivariate logistic analysis with backward logistic regression method has been performed including in the model variables showing significant (p<0.05) association with the dependent variable (low LnRHI) at the binary logistic analysis.

*p< 0.01; ^p< 0.05; a: per mHg; b: per mg/dl
